# Generation Victoria (GenV): Creating a large consented longitudinal infrastructure for discovery and intervention research

**DOI:** 10.23889/ijpds.v9i5.2821

**Published:** 2024-09-18

**Authors:** Melissa Wake, Sharon Goldfeld, Jatender Mohal, William Siero, Elizabeth Hughes, Susan Clifford, Simon Hall, Naomi Schwarz, Tony Frugier, Jing Wang, Suzanne Mavoa, Natasha Zaritski, Richard Saffery

**Affiliations:** 1 Murdoch Children's Research Institute

Designed to help solve complex problems facing children and adults, Generation Victoria is Australia’s largest-ever birth cohort, and the only such cohort mounted internationally during the COVID-19 pandemic. Here, we describe progress in bringing together this ‘cell-to-society’ consented cohort with diverse existing data and biosamples assets.

GenV is a whole-of-state consented cohort open to all Victorian children born October 2021-October 2023 and their parents. With over 117,000 participants to date (babies, mothers, fathers and non-binary parents), GenV closely reflects Australia's diverse demographics. At recruitment parents consent to GenV accessing (a) government managed and routinely collected administrative datasets across health, primary care, education, social care and geospatial and clinical datasets, (b) residual biosamples, (c) clinical and other service records and (d) spatial data.

To date, GenV has developed protocols, governance and technical requirements for linking birthing hospitals clinical data, state based administrative datasets and residual samples from statewide pathology labs. In pilot studies we have achieved high linkages rate (over 98%) with the state-based linkage spine and with clinical records from one birthing hospital. Universal newborn blood spot linkage is nearing completion. We are developing methods to include our natural and built environment exposome platform, antenatal imaging and trials data.

While challenging, GenV has encountered no significant barriers in creating an enduring, linked and secure data resource to advance future evidence-based decision making for child, family and adult outcomes. Our collaborative approach welcomes partnerships to co-build and enhance the muti-sectoral data and its utility.

